# Atopic Dermatitis: Sailing beyond the Sunset with a Multitude of Novel Treatments

**DOI:** 10.3390/jcm11123475

**Published:** 2022-06-16

**Authors:** Stamatios Gregoriou, Jacek C. Szepietowski

**Affiliations:** 1 1st Department of Dermatology-Venereology, Andreas Sygros Hospital, National and Kapodistrian University of Athens, 16121 Athens, Greece; 2 Department of Dermatology, Venereology and Allergology, Wroclaw Medical University, 50-367 Wroclaw, Poland; jacek.szepietowski@umw.edu.pl

Atopic eczema or dermatitis (AD) is a chronic pruritic inflammatory cutaneous disorder with an incidence up to 20% in children and 10% in adults depending on region and ethnicity. Due to severe pruritus, the chronicity of skin lesions, AD has been shown to have a vast psychosocial burden for patients. The AD patients report heavily decreased quality of life, raised stigmatization level and frequently develop secondary psychiatric comorbidities, such as depression and anxiety. The risk of suicidal thoughts and attempts is significantly increased [1,2]. Moreover, it is clear that not only the sick individual suffers but the disease affects also the family members [3]. Taking the above into consideration we are dealing with a common and important clinical problem. Understanding the AD pathogenesis in depth, developing new treatment options will definitely help holistic approaches to AD patients.

Atopic is a Greek adjective meaning literally “out of place” and teleologically “unexplained”. Eczema is a Greek noun meaning “due to boiling”. Dermatitis is also Greek, meaning cutaneous inflammation. Although we describe “Greek” only in the definition of Isocrates, who considered Greeks to be all those sharing in Greek education, Edward Perry was particularly insightful when asked by Coca and Cooke to help denominate the hypersensitivity to environmental allergens. A lot about the pathogenesis of atopic dermatitis still remains poorly illuminated. However, in this new era, rapid advances in basic science, dermatology and allergology research, along with several new therapeutic agents, set out a more optimistic approach for both clinicians and patients.

Epidermal components of AD pathogenesis include loss of function filaggrin gene polymorphisms; however, filaggrin variants are not the sole etiology for filaggrin downregulation in AD skin, and 40% of carriers of filaggrin null alleles never experience eczema. Filaggrin mutations have been reported to be associated with AD severity and persistence in adulthood, suggesting that filaggrin mutations represent a particular endotype [4]. The effect of Th2 inflammatory response on filaggrin downregulation and the normalization of filaggrin expression in patients treated with monoclonal antibodies or JAK-inhibitors is a field under exploration at this time. In addition, the impact of the new agents on other barrier-related proteins, such as involucrin and loricrin, should be part of an additional scope of investigation.

AD epidermis has decreased lipids, particularly ceramides, in both lesional and non-lesional skin as well as increased free fatty acids. IL-4 has been reported to downregulate ceramides synthesis via signaling through the STAT6 pathway. Alterations in the lipid composition of the epidermis can impair innate immunity and result in microbial superinfection in AD skin [5]. The role of the changes in the lipid microenvironment, such as Th2 inflammation via activation of CD1a antigen presenting proteins on Langerhans cells in AD, remains largely under-investigated. Although most moisturizers are beneficial in reducing the number of AD flares, evidence does not support that one moisturizer is better than another. In addition, despite early studies on the use of cleansers and moisturizers to prevent AD in infants and children reporting positive trends, more recent large-scale studies have failed to confirm these results. 

Genetic and acquired defects of tight junctions have been reported to play a crucial role in barrier dysfunction in AD patients. Decreased expression of claudin 1 has been associated with an increase in serum biomarkers of the Th2 driven response, suggesting cross-talk between the epithelial barrier and immunological inflammation in AD [6]. Areas of current investigation include the correlation between impaired tight junctions and antigen presentation, the interaction between claudin and filaggrin expression, and the effect of claudin expression on the proliferation and differentiation of keratinocytes.

Microbiota dysbiosis is emerging as an important feature of AD pathogenesis. *Staphylococcus aureus* is the principal pathogen that triggers the host immune system-related inflammation in the acute phase. In addition, the chronic persistence of *S. aureus* on eczematous skin lesions has been associated with the identification of staphylococcal biofilm communities on the skin of patients with AD. Recent reports suggest that *Staphylococcus epidermidis* might not be an innocent bystander but in certain conditions might also contribute to the inflammatory reaction in AD pathogenesis. *Cutibacterium acnes* enhances *S. aureus* cytolytic activity and the subsequent production of pro-inflammatory cytokines. Malassezia allergens can trigger a specific IgE response as part of AD pathogenesis. The role of *Candida albicans*, which has been found to often be part of the microbiota of AD, patients is not clear [7]. Novel therapeutic interventions, including probiotic and prebiotic preparations, as well as skin microbiota transplantation, are an emerging intervention to regulate microbiota dysbiosis, but evidence of their efficacy is still being evaluated.

AD is associated with food allergy, asthma, and allergic rhinitis and the gradual transition of one atopic disease into another has been denominated as the atopic march. Defining phenotypes and endotypes within the AD patient population in order to predict the future development of atopic march is an evolving effort. Although dupilumab is the only agent at this time that has an indication regarding AD, asthma, chronic rhinosinusitis with nasal polyposis, and eosinophilic esophagitis, the efficacy of the other novel therapeutic agents for AD, in the subpopulations of AD patients with other atopic comorbidities, is also a field of current clinical research interest.

The plethora of phenotypes and endotypes has raised investigational interest in the overlapping of AD and psoriasis, once considered to be two opposing immunopathogenic Th2 and Th1 disorders. Asian, pediatric, and intrinsic types of AD involve Th17, which is an important inflammatory pathway in psoriasis pathogenesis. As we investigate non-atopic comorbidities of AD, the shared patterns of comorbidities, such as metabolic syndrome or cardiovascular disorders, are considered, although evidence at this time is controversial. Clinical diagnosis, especially in children, might also be difficult as 20% of the cases might lack typical lesions and present a combination of both disease features, the so-called psoriatic eczema. In addition, plaque psoriasis restricted on the limbs might resemble nummular eczema while erythrodermic psoriasis might have an immunologic overlap of both Th17 and Th22 cells.

Animal model data could be helpful in understanding endotypes, phenotypes, and even the pharmacological properties of novel agents in a pre-clinical setting. The translational value of such data is still under debate, as there might be an overestimation of the potential efficacy of novel agents following encouraging results of in vivo studies in animal models. Published data of animal model characteristics might be helpful in selecting the appropriate model for a specific study purpose. The investigational aim should be to increase the predictability and translatability of animal model results to human clinical studies.

New developments of treatment modalities have recently resulted in the approval of first topical JAK-inhibitor—ruxolitinib cream for patients suffering from mild to moderate AD. This is a crucial step in topical AD therapy; for decades we only had the possibility of using topical corticosteroids and topical calcineurin inhibitors. With a good safety profile, ruxolitinib cream will definitely be of patients’ benefit, improving both pruritus and skin lesions [8,9]. Several more topical agents like tapinarof, difamilast, and roflumilast are also in advanced stage of development. Long term efficacy and safety of these formulations and comparison with current topical treatments are areas that are lacking data at this moment. 

EADV/EDF guidelines for the treatment of AD in patients’ candidates for systemic therapy suggest both classical systemic therapy as well as anti-IL4 and/or IL13 monoclonal antibodies and JAK-inhibitors as the first line treatment for the disorder. Even though the new agents provide long-term control of the disorder with a favorite safety profile, there are still matters under debate concerning their use, particularly from the view of the payers. The intermittent employment of the novel agents, transition from the higher dosage to the lower in JAK inhibitors, as well as class side effects, particularly in the light of the FDA warning, are questions still under discussion.

Studies have investigated the association of serum biomarkers for AD with treatment outcomes. An association does not necessarily allow prediction, and biomarkers have not currently been proven useful for patient stratification for systemic therapy for AD in published literature data. A recent consensus prioritized reliability, clinical validity, a high positive predictive value, prediction of the therapeutic response, and disease progression as potential biomarkers in AD and psoriasis [10]. Striving for ideal biomarkers in inflammatory cutaneous disorders is an ongoing effort, compromised by significant obstacles mainly associated with validity that does not allow the utilization of biomarkers in a way similar to their use in the disease of cancer. However, cost-effectiveness and the subsequent reimbursement of the novel agents could benefit considerably from the establishment of reliable biomarkers and are expected to drive further research in the field. Machine learning might offer significant insights in utilizing the available data of serum biomarkers.

Even as the present has revolutionized our view and treatment of AD, the future holds even more hope for the patients; multiple clinical trials and subpopulations analysis are currently underway. The main challenge at this time is to improve access to the novel therapies for an increased number of patients. Registries, biomarkers, and shared decisions by all stakeholders will be needed to attain this goal and even offer individualized treatment to AD patients. 

## References

[1] Kaaz K., Szepietowski J.K., Matusiak L. (2019). Influence of Itch and Pain on Sleep Quality in Atopic Dermatitis and Psoriasis. Acta Derm. Venereol..

[2] Dalgard F.J., Gieler U., Tomas-Aragones L., Lien L., Poot F., Jemec G., Misery L., Szabo C., Linder D., Sampogna F. (2015). The Psychological Burden of Skin Diseases: A Cross-Sectional Multicenter Study among Dermatological Out-Patients in 13 European Countries. J. Investig. Dermatol..

[3] Marciniak J., Reich A., Szepietowski J. (2017). Quality of Life of Parents of Children with Atopic Dermatitis. Acta Derm. Venereol..

[4] Furue M. (2020). Regulation of Filaggrin, Loricrin, and Involucrin by IL-4, IL-13, IL-17A, IL-22, AHR, and NRF2: Pathogenic Implications in Atopic Dermatitis. Int. J. Mol. Sci..

[5] Pavel P., Blunder S., Moosbrugger-Martinz V., Elias P.M., Dubrac S. (2022). Atopic Dermatitis: The Fate of the Fat. Int. J. Mol. Sci..

[6] Yoshida T., Beck L.A., De Benedetto A. (2022). Skin barrier defects in atopic dermatitis: From old idea to new opportunity. Allergol. Int..

[7] Hrestak D., Matijašić M., Paljetak H., Drvar D.L., Hadžavdić S.L., Perić M. (2022). Skin Microbiota in Atopic Dermatitis. Int. J. Mol. Sci..

[8] Papp K., Szepietowski J.C., Kircik L., Toth D., Eichenfield L.F., Leung D.Y., Forman S.B., Venturanza M.E., Sun K., Kuligowski M.E. (2021). Efficacy and safety of ruxolitinib cream for the treatment of atopic dermatitis: Results from 2 phase 3, randomized, double-blind studies. J. Am. Acad. Dermatol..

[9] Smith P., Yao W., Shepard S., Covington M., Lee J., Lofland J., Naim A., Sheth T., Parikh B., Yeleswaram S. (2021). Developing a JAK Inhibitor for Targeted Local Delivery: Ruxolitinib Cream. Pharmaceutics.

[10] Ziehfreund S., Tizek L., Hangel N., Fritzsche M., Weidinger S., Smith C., Bryce P., Greco D., Bogaard E., Flohr C. (2022). Requirements and expectations of high-quality biomarkers for atopic dermatitis and psoriasis in 2021—A two-round Delphi survey among international experts. J. Eur. Acad. Dermatol. Venereol..

